# A Rare Case of Late-Onset Pneumothorax and Bilateral Pulmonary Embolism Following Combined Liposuction and Mastopexy: A Case Report and Literature Review

**DOI:** 10.1093/asjof/ojaf062

**Published:** 2025-06-14

**Authors:** Mohamed Badie Ahmed, Fatima Saoud Al-Mohannadi, Albandare Abdulrahman Aldehaimi, Ghanem Aljassem, Abeer Alsherawi

## Abstract

Liposuction, although generally safe, carries risks of rare but serious complications, such as pneumothorax and venous thromboembolism, as illustrated in the case of a 48-year-old woman who developed bilateral pulmonary embolism and left pneumothorax 6 days after vibration amplification of sound energy at resonance–assisted liposuction with mastopexy, despite appropriate thromboprophylaxis. Presenting with dyspnea, chest pain, and leg swelling, computed tomography pulmonary angiography confirmed the diagnoses, which were successfully managed with anticoagulation and conservative measures, highlighting both the potential for delayed complication presentation and the limitations of current prophylaxis protocols. This case underscores the importance of extended postoperative vigilance, particularly for combined respiratory and thromboembolic events, and reinforces the need for meticulous surgical technique, including blunt-tip cannula utilization in thoracic-area procedures, as well as thorough patient counseling about warning signs, because even guideline-compliant prevention may not eliminate risks in susceptible individuals undergoing body contouring surgery.

**Level of Evidence:** 5 (Therapeutic)

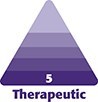

Liposuction is a widely performed cosmetic surgical procedure that is often combined with other interventions to enhance aesthetic outcomes.^1^ Although technological advancements have improved its safety profile, the procedure remains associated with rare but potentially life-threatening complications. Isolated liposuction can result in significant adverse events, including hematoma formation, pulmonary compromise, infection, and venous thromboembolism (VTE), with complication rates increasing when combined with other surgical procedures.^1^ Different liposuction techniques (eg, traditional, laser-assisted, vibration amplification of sound energy at resonance [VASER]) carry varying complication risks, which can range from minor to severe, including necrotizing soft tissue infections and even fatal outcomes.^2,3^

Among the most serious complications, pneumothorax—characterized by abnormal air accumulation in the pleural space—represents a critical though uncommon respiratory event. This complication may occur in various surgical contexts, including breast augmentation/reduction, abdominoplasty, intercostal nerve blocks, and rib graft harvesting. Etiology is multifactorial, encompassing patient-specific factors and iatrogenic causes such as inadvertent pleural injury during surgery, local anesthetic administration errors, or barotrauma.^4^ VTE remains another major concern, with an estimated incidence of 0.03% following liposuction, representing the procedure's leading cause of mortality.^1^ Significant independent risk factors include circumferential body contouring procedures, obesity, and hormone replacement therapy use.^5^ In this report, we present a case of simultaneous bilateral pulmonary embolism (PE) and pneumothorax following VASER-assisted liposuction with bilateral mastopexy, highlighting the critical importance of rigorous patient selection, individualized risk stratification, and thorough postoperative monitoring and immediate intervention when complications develop.

## CASE PRESENTATION

A 48-year-old woman with a history of gout and irregular intermenstrual bleeding managed with colchicine, allopurinol, and tranexamic acid presented to the emergency department 6 days after undergoing VASER-assisted liposuction of the abdomen and back with bilateral mastopexy performed in March 2025, reporting lower limb swelling, dyspnea, and nonradiating chest pain. The patient's surgical history includes a lower body lift performed in April 2024 and brachioplasty completed in January 2024. Additionally, she underwent laparoscopic sleeve gastrectomy in 2014. The patient was started on tranexamic acid approximately one and a half years ago. Initially, she was prescribed 500 mg 3 times daily, taken during her menstrual period for 5 days. After 1 year, the dose was increased to 1 g 3 times daily (total of 3 g/day). The patient underwent a wise-pattern breast mastopexy based on the superomedial pedicle technique, with 2 drains inserted. Concurrently, liposuction of the back and abdomen was performed. The back was infiltrated with 2500 mL of tumescent solution (comprising normal saline, adrenaline, and lidocaine), followed by VASER lipoemulsification for 9 min and 58 s. Fat aspiration was performed using spiral type, size 4 cannulas, yielding 850 cc of lipoaspirate. The abdomen was infiltrated with 1500 mL of the same tumescent solution, treated with VASER for 2 min and 46 s, and 850 cc of fat was aspirated using the cannulas. An additional drain was inserted at both the abdominal and back liposuction sites, bringing the total drains to 4 (2 for mastopexy, 1 for the back, and 1 for the abdomen).^6^ In addition, 1 g of intravenous tranexamic acid was administered intraoperatively to reduce the risk of bleeding. The surgery was uneventful under general anesthesia, and her calculated Caprini VTE score was 4, placing her at moderate risk for VTE. Thus, ambulation was encouraged (ambulated on postoperative Day 1), a below-knee pneumatic compression pump was applied, and thromboprophylaxis was prescribed during the inpatient period only (dalteparin 5000 IU daily). On postoperative Day 1, drain output was as follows: the breast drains demonstrated minimal serous output (right: 5 cc, left: 8 cc). The back drain showed significant serosanguinous output totaling 665 cc, whereas the abdominal drain produced 120 cc of serosanguinous fluid. Output from both the back and abdominal drains decreased significantly over the subsequent days, and all drains were removed on postoperative Day 3. Her hemoglobin declined from 11.1 g/dL preoperatively to 9.3 g/dL intraoperatively, then to 7.4 g/dL on postoperative Day 1, prompting transfusion of 1 unit of packed red blood cells, which raised her hemoglobin to 8.3 g/dL. The drop in hemoglobin is most likely attributable to blood oozing from the abdominal and back regions following liposuction. However, the patient remained hemodynamically stable and was discharged on postoperative Day 3 with standard follow-up instructions. She resumed her tranexamic acid doses after discharge.

**Figure 1.**
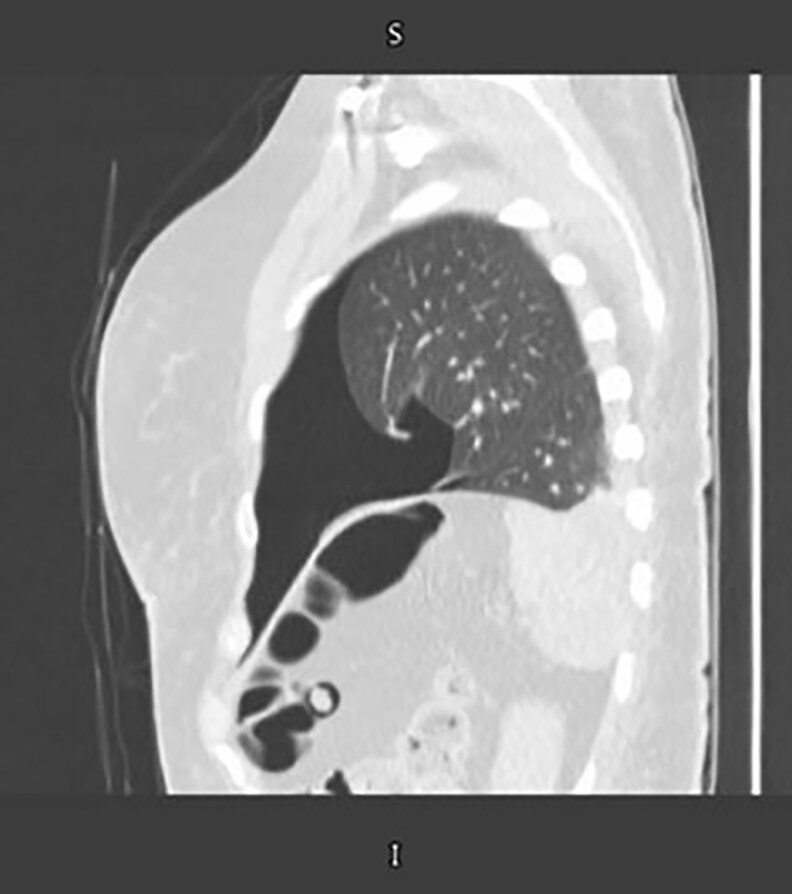
Computed tomography scan sagittal view showing a collapsed left lung in a 48-year-old female patient, confirming moderate pneumothorax.

**Figure 2.**
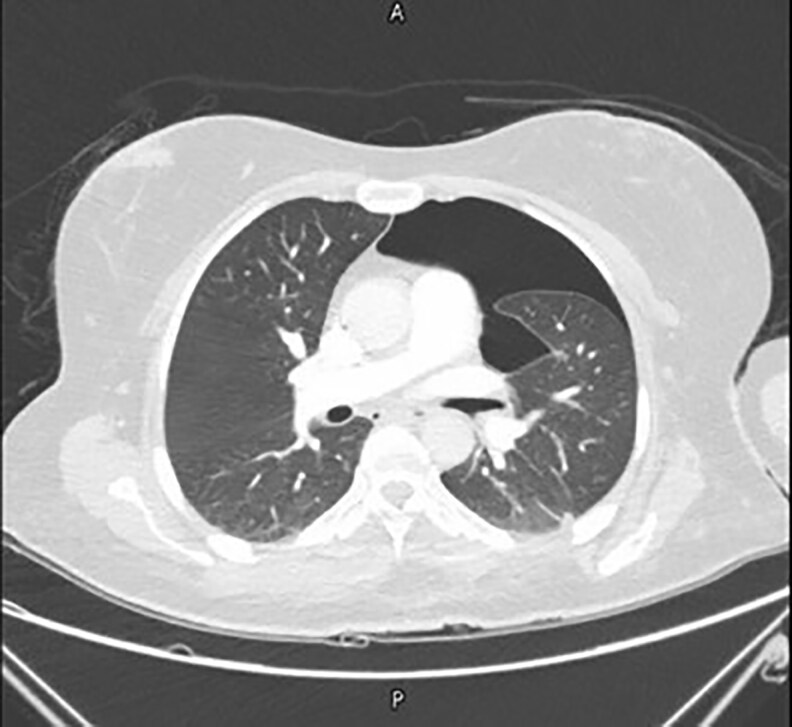
Computed tomography scan axial view showing a collapsed left lung in a 48-year-old female patient, confirming moderate pneumothorax.

Upon presentation to the emergency department (postop Day 6), physical examination was unremarkable, but computed tomography pulmonary angiography revealed bilateral segmental pulmonary emboli (right > left) and a moderate left pneumothorax with mild pleural effusion and lower lobe collapse (Figures 1, 2). She was admitted to the intensive care unit (ICU), started on therapeutic low-molecular-weight heparin, and managed conservatively for pneumothorax per cardiothoracic surgery because of clinical stability. Lower limb Doppler ultrasound (Day 2) showed no deep vein thrombosis (DVT), and surgical sites were intact, with mild ecchymosis but no hematoma/seroma. Over 3 ICU days, serial chest X-rays demonstrated improvement, with resolution of symptoms and normal room-air oxygenation with no active intervention performed (tube thoracotomy or percutaneous needle decompression). Anticoagulation was transitioned to apixaban, and she was discharged in stable condition after radiographic confirmation of full lung expansion. Follow-up was arranged with plastic surgery, anticoagulation, and gynecology clinics for medication review and further management.

## DISCUSSION

Liposuction remains a commonly performed day-case procedure in plastic surgery yet carries risks, including the rare but likely underreported complication of pneumothorax.^7,8^ This complication has been documented following various procedures, particularly when performed near the axillary region or posterior chest, with additional risk factors, including flexible infiltration cannula utilization and previous liposuction scar tissue.^9^ The American Society of Plastic Surgeons reports a pneumothorax incidence of ∼0.04% following liposuction, notably lower than the rates of DVT (0.2%-0.6%) and PE (0.3%) observed in suction-assisted abdominoplasty cases.^10^ A single-center review of 16,215 procedures found only 7 pneumothorax cases (1 per 2316 procedures), further highlighting its rarity.^11^ On the other hand, PE represents the most serious complication of liposuction, accounting for the highest procedure-related mortality with a 23% fatality rate. Although VTE risk in aesthetic surgery remains relatively low overall, body contouring procedures demonstrate the highest incidence. Key risk factors include advanced age and prolonged immobilization, among others.^10^

The present case of a 48-year-old female who developed bilateral PE and delayed-onset pneumothorax on postoperative Day 6 following VASER-assisted liposuction with bilateral mastopexy contributes novel insights when compared with the 12 cases of postliposuction pneumothorax summarized in Table 1.^7-12^ Several key distinctions and similarities warrant discussion. First, the delayed presentation of pneumothorax in our case (postoperative Day 6) appears unique among reported cases, which typically manifested either intraoperatively (*n* = 3), in the recovery room (*n* = 4), or within the first 24 to 48 h postoperatively (*n* = 5). This temporal pattern suggests our case may represent a distinct pathophysiological mechanism, possibly related to gradual air leakage from undetected pleural injury or delayed rupture of subclinical bullae. Notably, our patient lacked the pulmonary risk factors present in 3 reported cases (smoking history, asthma, or pneumonia), which, although reinforcing that pneumothorax can occur even without predisposing pulmonary conditions, their absence in this case underscores the likelihood that the pneumothorax was a surgical complication. The anatomical distribution of procedures also differed, as 6 reported cases involved axillary liposuction—a recognized risk factor—whereas our case involved abdominal/back regions only.^9,11^

**Table 1.** Summary of the 12 Cases of Postliposuction Pneumothorax

Author (year)	Age (years)	Sex	Medical history	Procedure	VTE prophylaxis	Presentation onset	Diagnosis	Management	Recovery period
Aguilar et al^10^	57	Female	Diabetes mellitus, hypertension, history of severe COVID-19 infection, BMI 34	Abdominoplasty, 360 liposuction	Compression stockings, intermittent pneumatic compression devices	First postoperative hours (pneumothorax), postoperative Day 4 (DVT, PE)	Right pneumothorax, DVT in both calves, right-sided PE, bilateral pulmonary fibrosis	Chest tube, enoxaparin 80 mg/12 h for 10 days and then with rivaroxaban 20 mg/day	5 weeks
Alwadai et al^9^	45	Female	Hypertension	Suction-assisted abdominoplasty, back liposuction, gluteal lipofilling	Hydration, early ambulation, compression stockings, enoxaparin 40 mg for 4 days	Postoperative Day 1	Left pneumothorax	Chest tube	4 days
Ansari et al^12^	54	Female	Endometriosis, anxiety, depression, active tobacco utilization (40 pack-year history)	Abdominal liposuction	—	12 h postoperative	Right pneumothorax, fat embolism syndrome	Chest tube	2 days
Nasr et al^7^	60	Male	Rheumatoid arthritis, paroxysmal atrial fibrillation, multiple lumbar surgeries	Suction-assisted abdominoplasty	—	Postoperative Day 1	Bilateral pneumothorax, pneumomediastinum, pneumoperitoneum	Pigtail catheters for the pneumothorax, pneumoperitoneum managed conservatively	12 days
Taha and Tahseen^8^	47	Female	Bronchial asthma during adolescence, pneumonia 6 months before the procedure	Abdominoplasty, 360 liposuction, breast reduction	Compression stockings, enoxaparin 40 mg for 4 days	Postoperative Day 1	Left pneumothorax	Chest tube	5 days
Mentz et al^11^	46	Female	—	Liposuction of arm, flank, knee, and inner thigh	—	Recovery room	Left pneumothorax	Observation	—
Mentz et al^11^	53	Male	—	Liposuction of abdomen, flank, chest, and axilla	—	Recovery room	Left pneumothorax	Chest tube	—
Mentz et al^11^	41	Female	—	Liposuction of back, flank, and axilla	—	Intraoperatively	Left pneumothorax	Chest tube	—
Mentz et al^11^	55	Female	—	Liposuction of abdomen, flank, hip, pubic, back, and axilla	—	Intraoperatively	Right pneumothorax	Chest tube	—
Mentz et al^11^	35	Female	—	Liposuction of arm, flank, outer and inner thigh, back and axilla	—	Recovery room	Right pneumothorax	Chest tube	—
Mentz et al^11^	39	Female	—	Liposuction of arm, flank, abdomen, inner and outer thigh, back, and axilla	—	16 h postoperatively	Left pneumothorax	Observation	—
Mentz et al^11^	50	Female	—	Liposuction of arm, abdomen, flank, inner thigh, and axilla	—	Intraoperatively	Right pneumothorax	Chest tube	—

DVT, deep vein thrombosis; PE, pulmonary embolism; VTE, venous thromboembolism.

The co-occurrence of bilateral PE with pneumothorax in our patient mirrors 3 cases in the series that featured concurrent thromboembolic events. However, our case uniquely received appropriate VTE prophylaxis (early ambulation, below-knee pneumatic compression pump, and thromboprophylaxis [dalteparin]), suggesting that even guideline-compliant prevention may not eliminate risk in susceptible individuals. The conservative management of pneumothorax in our patient aligns with only 2 other cases in the series, both of which had favorable outcomes. This supports current recommendations for a conservative approach in stable patients.^8^ The 2 to 5 day recovery period in our case compares favorably with the wider range (2 days to 5 weeks) observed in the series, where prolonged courses typically involved complex presentations (eg, Case 1 with bilateral pulmonary fibrosis). Demographically, our patient's age (48 years) falls within the reported range (35-60 years), supporting the observation that age may not be a primary risk determinant. The absence of significant comorbidities in our patient contrasts with several cases featuring conditions, such as diabetes, hypertension, or rheumatoid arthritis, suggesting that pneumothorax risk may be more procedure related than patient dependent. Although rare, pneumothorax may occur secondary to pulmonary embolism, particularly in cases where the presentation of pulmonary symptoms is delayed.^13^ Notably, combined procedures did not appear to increase risk in either our case or the series (4/12 cases involved combined procedures).

These findings collectively underscore 3 critical implications for practice: (1) pneumothorax vigilance should extend beyond the immediate postoperative period, particularly for thoracic/axillary procedures; (2) combined VTE/pneumothorax presentations require comprehensive evaluation despite prophylaxis; and (3) conservative management may be appropriate for stable pneumothorax cases. Future research should investigate whether VASER-assisted techniques modify complication profiles compared with traditional liposuction. Current evidence recommends several technical precautions to minimize liposuction-related complications. These include exclusive utilization of blunt-tipped cannulas, maintaining continuous tactile awareness of the cannula position by keeping it palpable within the subcutaneous tissue using the opposite hand and utilizing stiff infiltration cannulas (>3.5 mm diameter) to reduce pleural injury risk.^14^ Additional measures involve minimizing positive pressure ventilation, exercising particular caution in the axillary region, and avoiding unnecessary patient repositioning during procedures.^9^ Importantly, comprehensive patient education forms a critical component of risk mitigation. This includes obtaining thorough informed consent that details potential complications, as well as providing clear instructions about warning signs to facilitate early recognition and prompt treatment.^15^ Furthermore, it is important to highlight that numerous high-quality studies and meta-analyses have demonstrated that the utilization of tranexamic acid—whether administered preoperatively, intraoperatively, or postoperatively—is not associated with an increased risk of thromboembolic events in aesthetic procedures.^16-18^

## CONCLUSIONS

Liposuction remains a widely sought-after cosmetic procedure, but it requires meticulous technique and patient assessment to minimize risks, particularly life-threatening complications, such as pneumothorax and PE. Surgeons must prioritize safety protocols, proper patient selection, and postoperative monitoring, while maintaining a high index of suspicion to promptly detect abnormal signs and symptoms in the early postoperative period—because rapid recognition and intervention are keys to preventing devastating outcomes.
